# Beam size limit for pencil minibeam radiotherapy determined from side effects in an *in-vivo* mouse ear model

**DOI:** 10.1371/journal.pone.0221454

**Published:** 2019-09-04

**Authors:** Matthias Sammer, Katharina Teiluf, Stefanie Girst, Christoph Greubel, Judith Reindl, Katarina Ilicic, Dietrich W. M. Walsh, Michaela Aichler, Axel Walch, Stephanie E. Combs, Jan J. Wilkens, Günther Dollinger, Thomas E. Schmid

**Affiliations:** 1 Institut für Angewandte Physik und Messtechnik (LRT2), Universität der Bundeswehr München, Neubiberg, Germany; 2 Department of Radiation Oncology, Technical University of Munich, Klinikum rechts der Isar, Munich, Germany; 3 Institut für innovative Radiotherapy (iRT), Department of Radiation Sciences (DRS), Helmholtz Zentrum München (HMGU), Oberschleißheim, Germany; 4 Deutches Konsortium für Translationale Krebsforschung (DKTK), Partner Site Munich, Munich, Germany; 5 Research Unit Analytical Pathology, Helmholtz Zentrum München, German Research Center for Environmental Health, Oberschleißheim, Germany; Technische Universitat Dresden, GERMANY

## Abstract

Side effects caused by radiation are a limiting factor to the amount of dose that can be applied to a tumor volume. A novel method to reduce side effects in radiotherapy is the use of spatial fractionation, in which a pattern of sub-millimeter beams (minibeams) is applied to spare healthy tissue. In order to determine the skin reactions in dependence of single beam sizes, which are relevant for spatially fractionated radiotherapy approaches, single pencil beams of submillimeter to 6 millimeter size were applied in BALB/c mice ears at a Small Animal Radiation Research Platform (SARRP) with a plateau dose of 60 Gy. Radiation toxicities in the ears were observed for 25 days after irradiation. Severe radiation responses were found for beams ≥ 3 mm diameter. The larger the beam diameter the stronger the observed reactions. No ear swelling and barely reddening or desquamation were found for the smallest beam sizes (0.5 and 1 mm). The findings were confirmed by histological sections. Submillimeter beams are preferred in minibeam therapy to obtain optimized tissue sparing. The gradual increase of radiation toxicity with beam size shows that also larger beams are capable of healthy tissue sparing in spatial fractionation.

## Introduction

Radiotherapy (RT) is one of the most widely used forms of treatment in cancer therapy. The goal of radiotherapy is to cure the tumor by maximizing the dose within the tumor while simultaneously maintaining the side effects in the healthy tissue at the lowest possible level. The radiation damage in the healthy tissue surrounding the tumor is the main limit in RT. Reduced side effects at reasonable tumor control were achieved by the development of e.g. fractionation and intensity modulated radiotherapy (IMRT), which both are used in modern treatment modalities. However, radiation toxicities are still an issue in RT.

A novel method to spare healthy tissue and therefore increase the therapeutic window is spatial fractionation. Sub-millimeter sized pencil or planar beams (hereafter: minibeams) are applied in a grid pattern (pencil beams) or regularly spaced (planar beams) to irradiate a tumor. As the inter-beam distances are chosen larger than the beam diameter, a channel-like dose pattern is produced with enhanced dose maxima leaving a large fraction of the total irradiated area of the healthy tissue unirradiated, thus increasing the overall dose tolerance of the tissue and reducing side effects. Overall, spatial fractionation provides more treatment flexibility.

While spatial fractionation can be applied using x-ray planar microbeams (x-ray microbeam radiation therapy (MRT)) or x-ray minibeam radiation therapy [1–3], also proton or heavy ion minibeams can be spatially fractionated and may have even more advantages, especially due to their limited range in depth [4,5]. The irradiation modes differ in the dose deposition in the target volume. By applying low (100s of keV) energy x-ray micro- or minibeams, the planar channel structure, i.e. the inhomogeneous dose pattern (with peaks and valleys) remains basically unchanged in the tumor. Not all tumor cells receive a lethal dose if the tumor is irradiated from one direction. Nevertheless, some specific tumors can be controlled using a channel irradiation due to particularly sensitive tumor vessels [6]. Alternatively, a homogeneous dose distribution is obtained only by interlacing x-ray minibeams from two or more directions but will require a sub-millimeter precision to adjust the interlacing fields [7].

In proton or ion minibeam therapy, additional options are available to obtain a homogeneous dose distribution in the tumor due to the finite range and the lateral scattering of the ion beams. One option is to irradiate from only one side such that the minibeams overlap within the target volume due to their lateral spread on their way into the tumor where the ions are stopped in the Bragg peak [8,5]. An adjustment of the interbeam spacing according to the depth of the tumor is required in order to obtain a homogeneous dose distribution in the target volume while sparing as much healthy tissue as possible by spatial fractionation. The various pencil and planar proton beam geometries are calculated in dependence of the depth and the size of the tumor in [9]. If heavy ions such as carbon or oxygen are used, the sub-millimeter beam size remains unchanged many centimeters into the tissue similar to x-ray minibeam therapy [4]. Therefore, smaller inter beam distances are required to obtain a homogeneous tumor dose. The higher RBE of heavy ions may provide additional advantages for tumor control. As a second option, the limited range of ions allows for interlacing minibeams even from opposite directions.

Despite the differences in the irradiation of the target volume, the basic principles involved in sparing of healthy tissue by spatial fractionation are the same for x-ray, proton or heavy ion minibeams. However, the underlying mechanisms of the normal tissue sparing effects are not yet fully understood. The dose volume effect [10], which expresses the volume dependence of the dose that causes a certain effect (or its probability) in a volume [11], is assumed to play a key role for the higher dose resistance. If small volumes are considered, the tissue repair by proliferating and migrating cells is more efficient than in bigger volumes but this requires more work to verify. Additional effects such as the fast capillary repair [12] or the inherent resistance of capillaries to high doses [13] might also be important for the mechanistic explanation of high dose resistances seen in spatially fractionated tissues.

Due to the lack of clinical and experimental data on minibeams, it is still hard to predict the outcomes, advantages and disadvantages of such applications. The variety of beam configurations leaves a lot of opportunities to be investigated. While planar shaped beams are mostly used in MRT, pencil beams can be used in ion or proton minibeam therapy, which may offer enhanced tissue sparing capabilities in comparison to planar minibeams. A theoretical investigation of different geometrical settings on a cell survival basis of proton minibeams has found benefits for pencil proton minibeams in comparison to planar proton minibeams [9]. Nevertheless, it is not only the shape of the beams which needs to be considered. The dose pattern is a result from the beam shape, the beam sizes and the grid pattern. The healing process and thus the radiation response depends on all of the mentioned parameters. The total effect of an irradiation pattern has been investigated in experimental data when several beams were set side by side for spatial fractionation. In order to model and predict the radiation responses of sub-millimeter beam patterns, one needs to know the effects of the single beams alone. Dilmanian et al. found a strong sparing effect concerning long-term paralysis, weighed and rotarod test rotarod (rotating rod; measures balance, coordination and overall physical condition) for the minibeam irradiated spinal cord of rats with planar beams of up to 0.68 mm beam size and 4 mm beam distance [7]. Prezado et al., however, showed that 0.68 mm are not a general limit, as they found a strong sparing effect for planar proton minibeams in the size of 1.1 mm in irradiated rat brains [14]. A major question however still remains unanswered, whether an absolute beam size limit for tissue sparing exists and how the radiation response proceeds after such a limit.

Here, we apply single photon pencil beams of various diameters in an *in-vivo* mouse ear model. The influence of beam size to side effects could be investigated without disturbance of adjacent beams, which are usually present for spatially fractionated radiotherapy approaches. The resulting acute side effects were monitored for 25 days post-irradiation. The data provide detailed information on the size dependent reactions of single pencil beams, which will be important to predict side effects in spatially fractionated minibeam treatments.

## Materials and methods

### Irradiation conditions

To investigate the side effects of single small beams on the skin as one of the healthy tissues irradiated in radiotherapy, a mouse ear model of BALB/c mice without a tumor was used. The right ears of the mice were irradiated in one fraction by circular x-ray beams between 0.5–6 mm in diameter and a plateau dose of 60 Gy. The irradiations were carried out with a Small Animal Radiation Research Platform (SARRP, Xstrahl Ltd., Camberley, Surrey, UK) at the Klinikum rechts der Isar, Munich, Germany. The x-ray tube operated at a voltage of 70 kV (broad focal spot, filtered by 1 mm Al) and the current setting was 30 mA. Seven different brass collimators were used to form the desired radial field sizes at the ear of 0.5, 1, 2, 3, 4, 5 and 6 mm in diameter. The distance from the source (focal spot) to the ear was kept at 35 cm and the distance from collimator to ear was kept at 5 cm for all collimators used. A self-made mouse holder with an additional platform to fix the ear was used during the irradiation as in [8]. A Perspex area was embedded at the ear location in the holder for a controlled dose application due to the similar scattering and absorption of the x-rays as in water. To mark the irradiation and ear position, a Gafchromic® EBT3 film was placed behind the ear. The films, where both the contour and the irradiated field was marked, could be used for the determination of the irradiation field in histological sections.

### Animal model and ethical approval

Female, 8 to 12 week old BALB/c mice (Charles River Laboratories, Sulzfeld, Germany) were exposed to a 12-hr light/dark cycle at the temperature-regulated animal facility of the Klinikum rechts der Isar, Munich, Germany. The animals had *ad libitum* access to food and water. The experiment was prospectively approved by the District Government of Upper Bavaria (Regierung von Oberbayern, 80534 München, Germany; approval number: 55.2-1-54-2532-144-13) and performed in accordance with the Animal Welfare and Ethical Guidelines of the Klinikum rechts der Isar, Munich, Germany.

The ears of the mice were targeted as the region of interest in our experiment due to the lack of pigmentation and the large ear size (~ 1 cm in diameter). The thin ears (~ 220 μm) ensure a homogeneous depth dose distribution as absorption is small for 70 keV x-rays (attenuation coefficient μ in water ~ 0.2 cm^-1^ [15]).

For a 25 days follow-up study, 4 BALB/c mice for each group were irradiated with 0.5, 1 or 2 mm field size and 3 BALB/c mice for each group were irradiated with 3, 4, 5 or 6 mm field size. The total number of 24 mice was distributed over 7 different beam size groups to create a meaningful trend line rather than distinguish the single groups with a high statistical significance.

Irradiation was performed under general anesthesia, induced by intraperitoneal injection of medetomidine (0.5 mg/kg), midazolam (0.5 mg/kg) and fentanyl (0.05 mg/kg). The antagonist atipamezole (2.5 mg/kg), flumazenil (0.5 mg/kg) and naloxone (1.2 mg/kg) was administered subcutaneously at the latest 45 minutes after induction of the anesthesia. Euthanasia was perfomed by cervical dislocation.

### Dosimetry and field characterization

**Fig 1:**
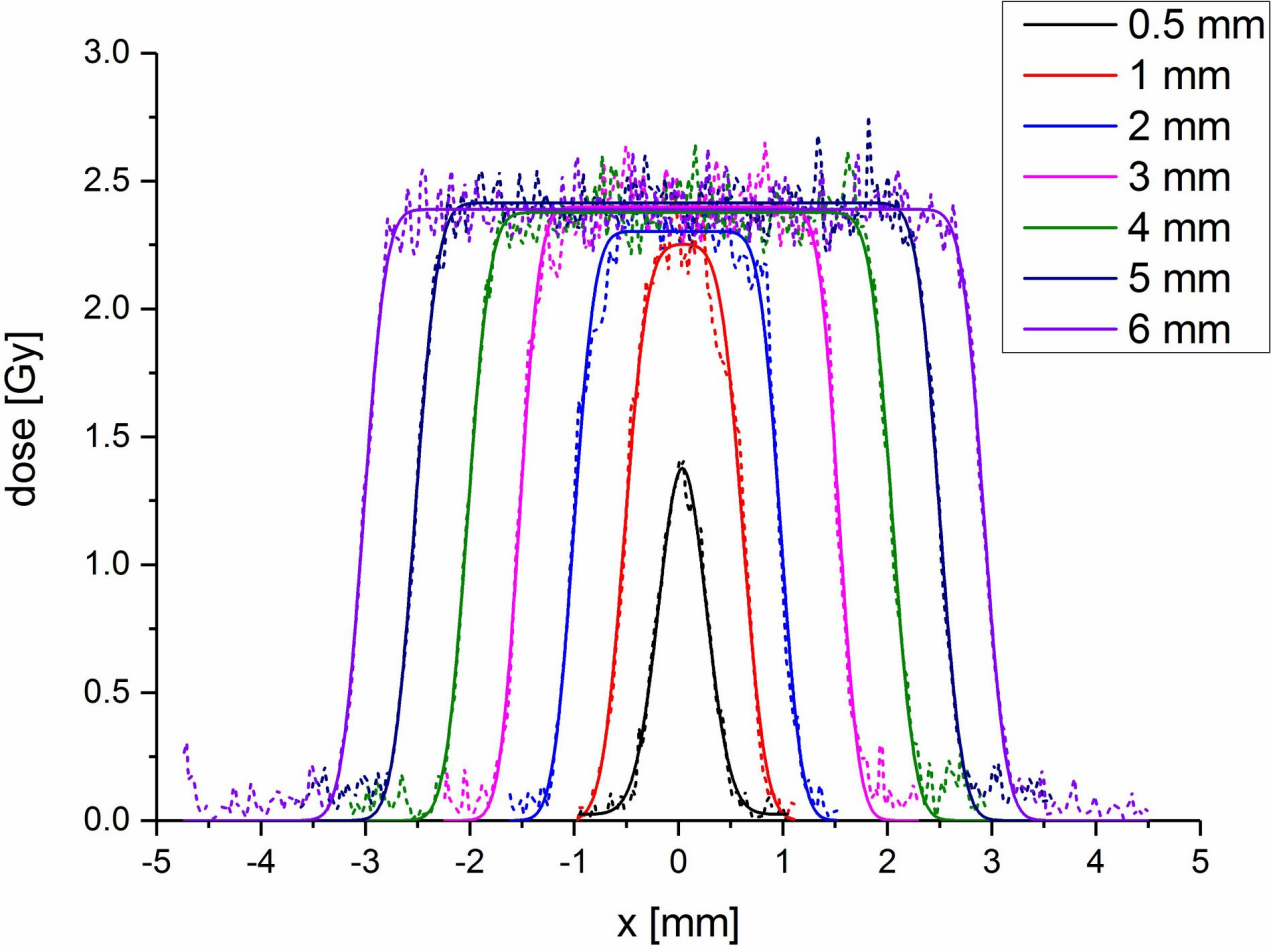
Dose profiles for the collimators used. The dashed lines show the measured values at 1200 dpi. The solid lines are fits using Eq (1) except for the smallest beam size, where a Gaussian approximation is used.

To harden the beam of the 70 kV x-ray source a 3 mm aluminum filter was used. The dose rate of the resulting beam was determined in an open 5 cm x 5 cm field at the SARRP at the surface of a 20 x 2 x 2 cm^3^ solid water slab using a calibrated ionization chamber (IC TM23342, PTW, Freiburg, Germany). The absolute dose rate of the source allowed for the calibration of a Gafchromic EBT3 film (GafChromic^TM^, Ashland, US) batch. The radiochromic films were necessary since the small fields of the collimators had to be characterized and the ionization chamber was not suitable for these small fields. Film processing was done according to Reinhardt et al. [16]. For every collimator, an EBT3 film of the same batch (Lot# 03171401) as the calibration curve was irradiated with one minute exposure time at the location of the mouse ear. Three dose profiles were extracted out of the EBT3 film. A representative measured dose profile (scanned at 1200 dpi) for every collimator is plotted in Fig 1 and fitted with a sum of two error functions defined as

$$D(x)=D_{p}\times \left(erf(\frac{x-x_{low}}{\sqrt{2}\sigma })-erf(\frac{x-x_{high}}{\sqrt{2}\sigma })\right),$$ (1)

where x_low_ and x_high_ are the half maximum positions, D_p_ in Gy is the plateau height and σ is the slope width. The smallest beam diameter was fitted with a Gaussian of standard deviation σ. The resulting mean values of the full width half maximum FWHM, the fit parameter σ as well as the dose rate $\dot{D}$ (D_p_ divided by exposure time) are presented in Table 1.

**Table 1:** Determined parameters for the different collimators. Measured values of the different dose rates, field sizes and slope widths (or Gaussian σ) for the different collimators at the location of the mouse ear. The dose rate for the smallest beam was measured at the maximum of the Gaussian fit. The uncertainties in dose rate, field size and edge sharpness are the standard deviations of the fits for the three individual profiles.

Collimator diameter [mm]	D˙[Gymin]	Field size FWHM [mm]	Fit parameter σ [mm]
6	2.37 ± 0.012	5.947 ± 0.021	0.147 ± 0.021
5	2.42 ± 0.012	5.033 ± 0.006	0.150 ± 0.020
4	2.37 ± 0.012	4.073 ± 0.016	0.153 ± 0.016
3	2.39 ± 0.012	3.053 ± 0.012	0.153 ± 0.026
2	2.32 ± 0.02	1.963 ± 0.006	0.167 ± 0.031
1	2.25 ± 0.04	1.130 ± 0.010	0.169 ± 0.023
0.5 (GAUSSIAN)	1.29 ± 0.08	0.53 ± 0.07	0.227 ± 0.026

The measured field sizes are in good agreement with the desired field sizes. The dose rates in the plateau are the same within the given uncertainty values besides the smallest diameter, where the dose rate dropped to nearly half the other values. The absolute dose accuracy was estimated to be within 10% which delivers a dose interval of 60 ± 6 Gy.

### Ear thickness measurements

For a follow-up period of 25 days, the thickness of the treated right ear and the untreated left ear were measured every 2–4 days in triplicate using an electronic external measuring gauge (C1X079, Kröplin GmbH, Schlüchtern, Germany), with measuring contacts of 6 mm in diameter. The statistical errors were corrected by the sudent´s t-distribution with a confidence interval of 65%.

### Skin reaction scoring

Acute skin reactions were monitored with regard to erythema and desquamation. The scores erythema and desquamation were added up to a total skin score (Table 2). The systematic error was estimated to be 0.5 and the statistical error was corrected by the student´s t-distribution with a confidence interval of 65%.

**Table 2:** Skin Score table. The total skin score is defined as the sum of both, the erythema scale and the desquamation scale (table adapted from Girst et al. [8]).

Erythema	Scale	Desquamation	Scale
no	0	no	0
mild	0.5	dry	1
definite	1.5	crust formation	2
severe	3	moist	3

### Histology

After the follow-up period, mice were sacrificed only on day 25 after irradiation, since it is the expected time point of the maximum reaction according to Girst et al. [8]. The ears were dissected, formalin-fixed and paraffin embedded. Tissue sections were cut (2 μm thickness) and stained with hematoxylin and eosin (H&E) for microscopic examination. To verify the irradiation field, the Gafchromic films with the marked ear outlines as well as the irradiation fields were used.

## Results

**Fig 2:**
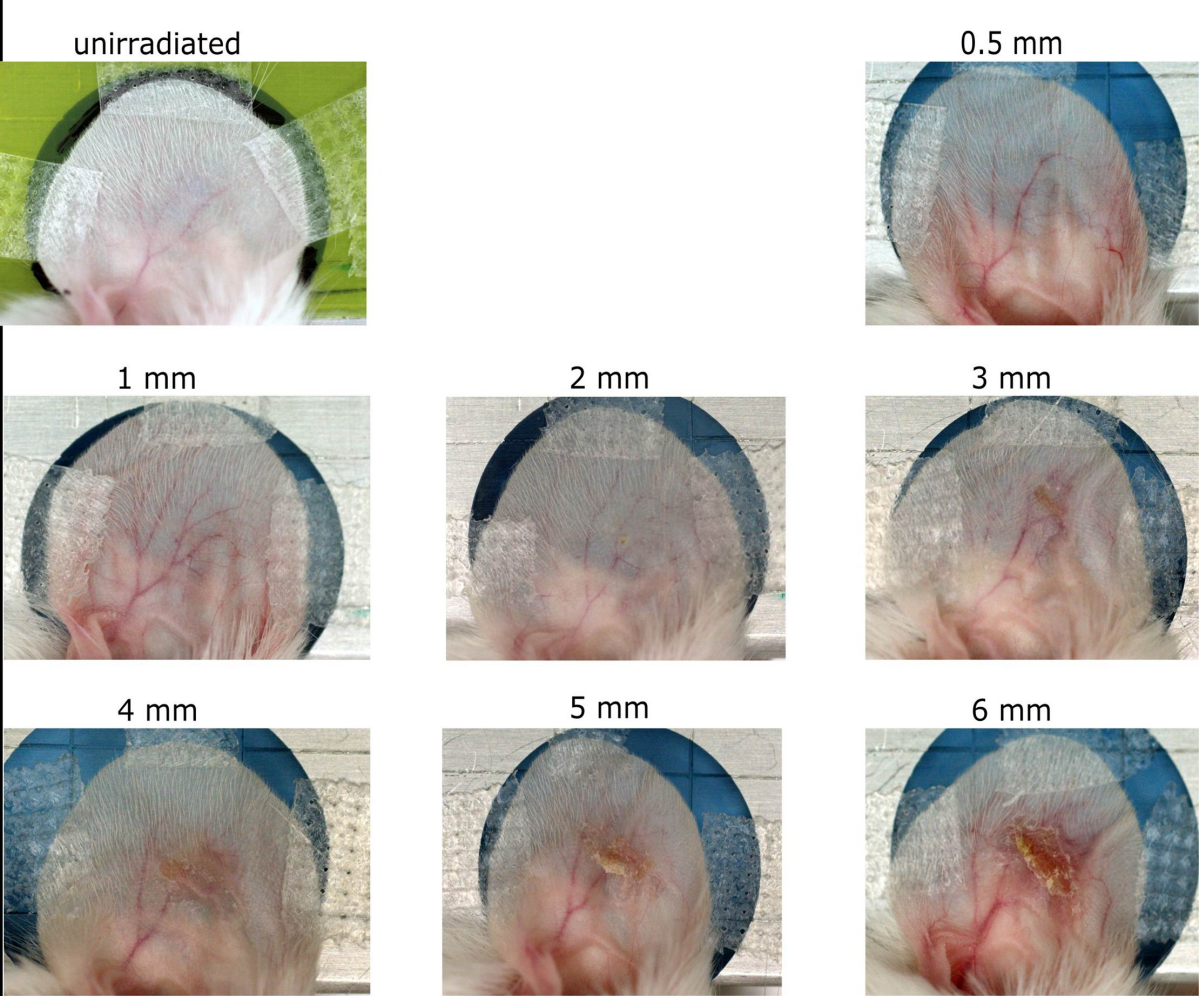
Photos of the irradiated ears on day 25 post-irradiation. The photo for the unirradiated ear was taken on day 0 since no control group is available. The softer contrast in this picture is due to the Gafchromic film underneath the ear.

The mice were monitored and observed for 25 days post-irradiation, which is the expected time point of the maximum skin reaction found in the previous study of Girst et al. [8]. For the first impression, photo documentation of the ears was performed as baseline before irradiation on day 0 (as a control) and the expected day of the maximum reaction on day 25 for comparison. A representative picture of every group is shown in Fig 2.

A strong correlation between skin response and irradiated field size is observed. Especially the crust formation is remarkable for the larger fields. An increase of the erythema with beam size is also observable. However, as mice were anaesthetized and fixed for a better picture quality, the decreased blood supply weakens the reddening.

### Skin response scoring

**Fig 3:**
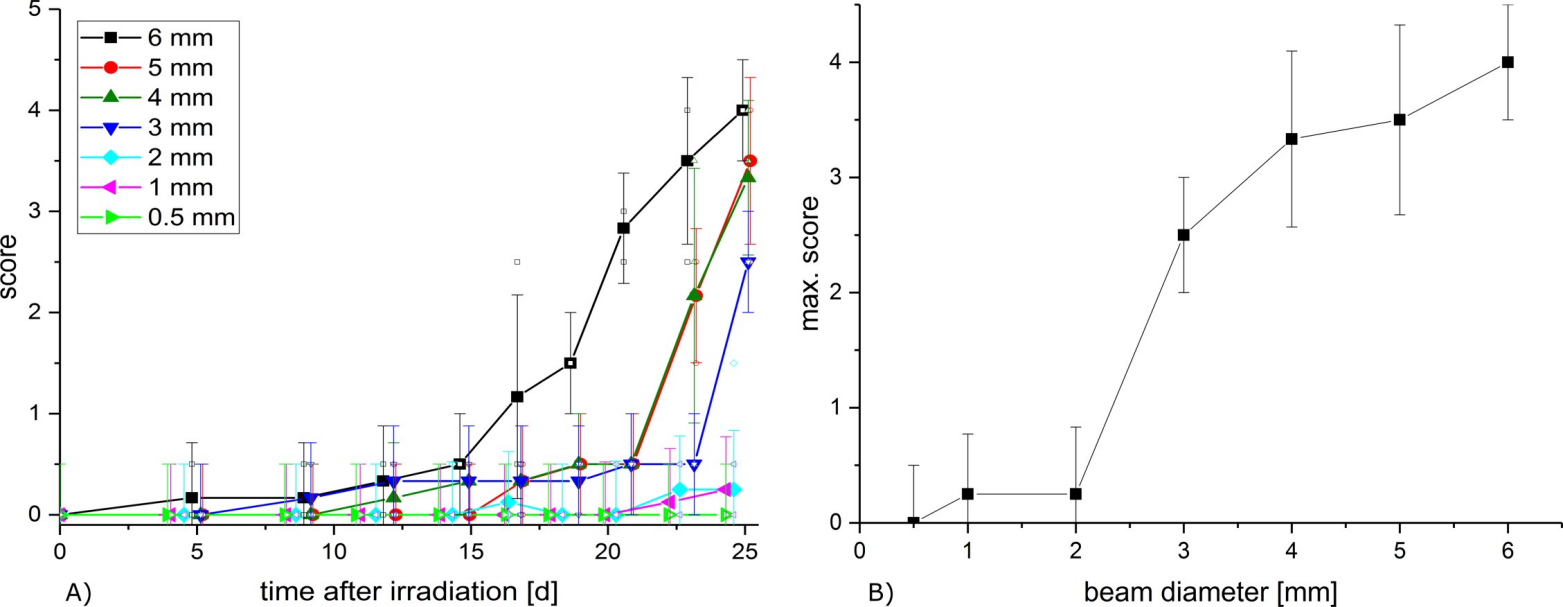
Measured scores. A) Mean score over time (sum of erythema and desquamation; mean ± standard error of the mean SEM). B) Mean score ± SEM over beam diameter on day 25, which was found as the day of the maximum score by Girst et al. [8].

The scoring and quantification of erythema and desquamation according to Table 2 was conducted in intervals of 2–4 days up to 25 days after irradiation. The total score, defined as the sum of erythema and desquamation, is plotted in Fig 3A. The data from the previous study of Girst et al. [8], where the same mouse strain was irradiated with a square field of 7.2x7.2 mm^2^ also applying a 60 Gy dose, fits very well with the observations in this work.

In comparison to the unirradiated left ears (constant score 0), a significant change cannot be observed for irradiated fields ≤ 2 mm. All fields larger than 2 mm show a definite skin response. Fig 3A also shows that the smaller the irradiation field, the later the skin response and the smaller the maximum score. While the 4 mm and 5 mm fields are almost indistinguishable despite their quite large error bars, the 6 mm beam shows the strongest skin response.

The maximum score is observed on day 25 and is plotted over beam size in Fig 3B.

The skin responses of the three smallest irradiation fields barely yielded a measurable skin score (≤ 0.25). A strong increase of the reaction can be detected from field diameters D > 2 mm until the biggest field diameters of 6 mm. A maximum observed reaction of ~ 4.5 was found in the previous study of Girst et al. [8] and extends Fig 3B nicely.

### Measurement of ear thickness

**Fig 4:**
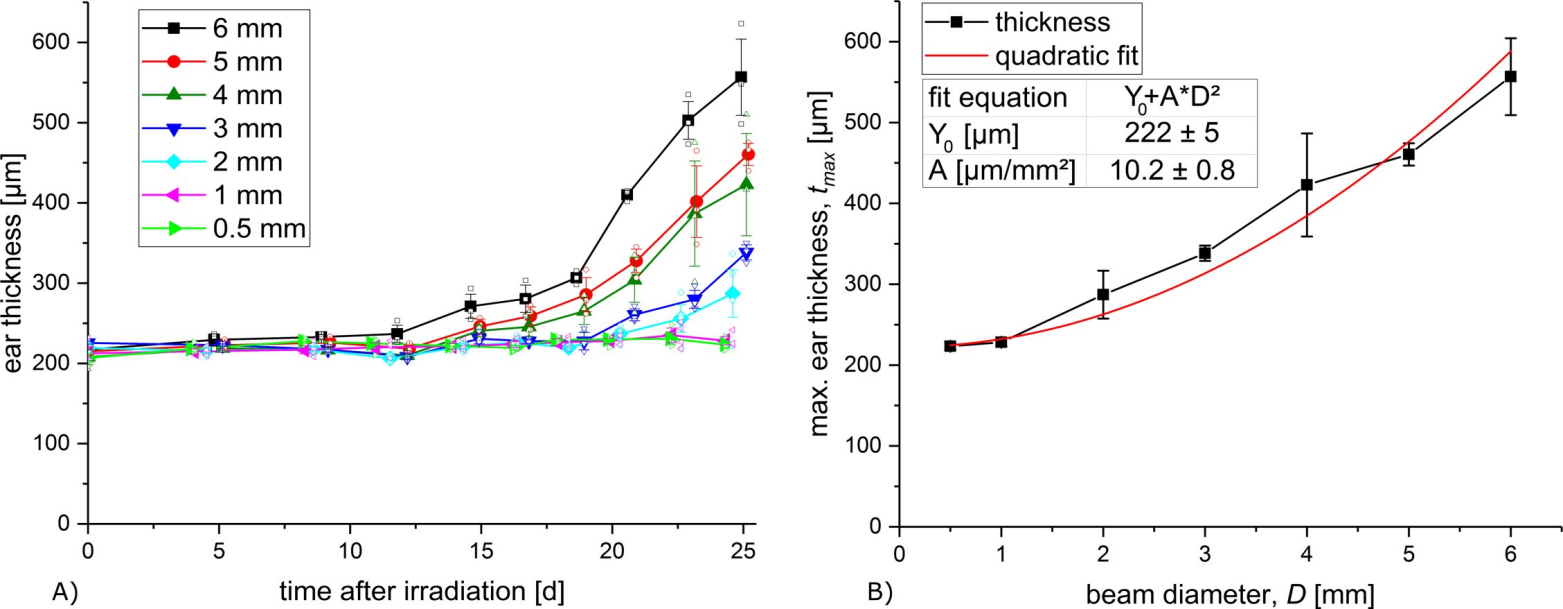
Measured ear thickness. A) Ear thickness (mean value ± SEM) of the irradiated ear over the monitored time span. B) Maximum ear thickness (mean ± SEM) over beam diameters.

The thickness measurements were carried out at the same time points as the scoring. The mean thickness is plotted over time after irradiation in Fig 4A.

Over the 25-day monitoring period, no increase of the ear thickness could be detected in the 0.5 and 1 mm group. All fields ≥ 2 mm led to an increase in thickness. The earliest measured increase in swelling was recorded for the 6 mm field. The smaller the fields, the later the onset of the swelling reaction was observable. The strongest swelling in this study was measured for the largest field with an average ear thickness of ~ 550 μm (initial ear thickness: ~ 200 μm) for the 6 mm diameter field. It has previously been shown that a further increase in ear swelling is observed for even larger irradiation as the used 7.2 mm × 7.2 mm (8.12 mm radius equivalent) by Girst et al. [8] with a maximum observed ear thickness of ~ 862 μm.

The maximum ear thicknesses are plotted over the field sizes in Fig 4B.

A strong quadratic correlation t_max_ = Y_0_ + AD^2^ between maximum ear thickness t_max_ and irradiated beam diameter D is observed with an R-square of 0.97. The fit parameters are given in Fig 4B. A saturation effect might appear if larger irradiation fields are applied, but was not detected within the used field sizes.

### Histology

**Fig 5:**
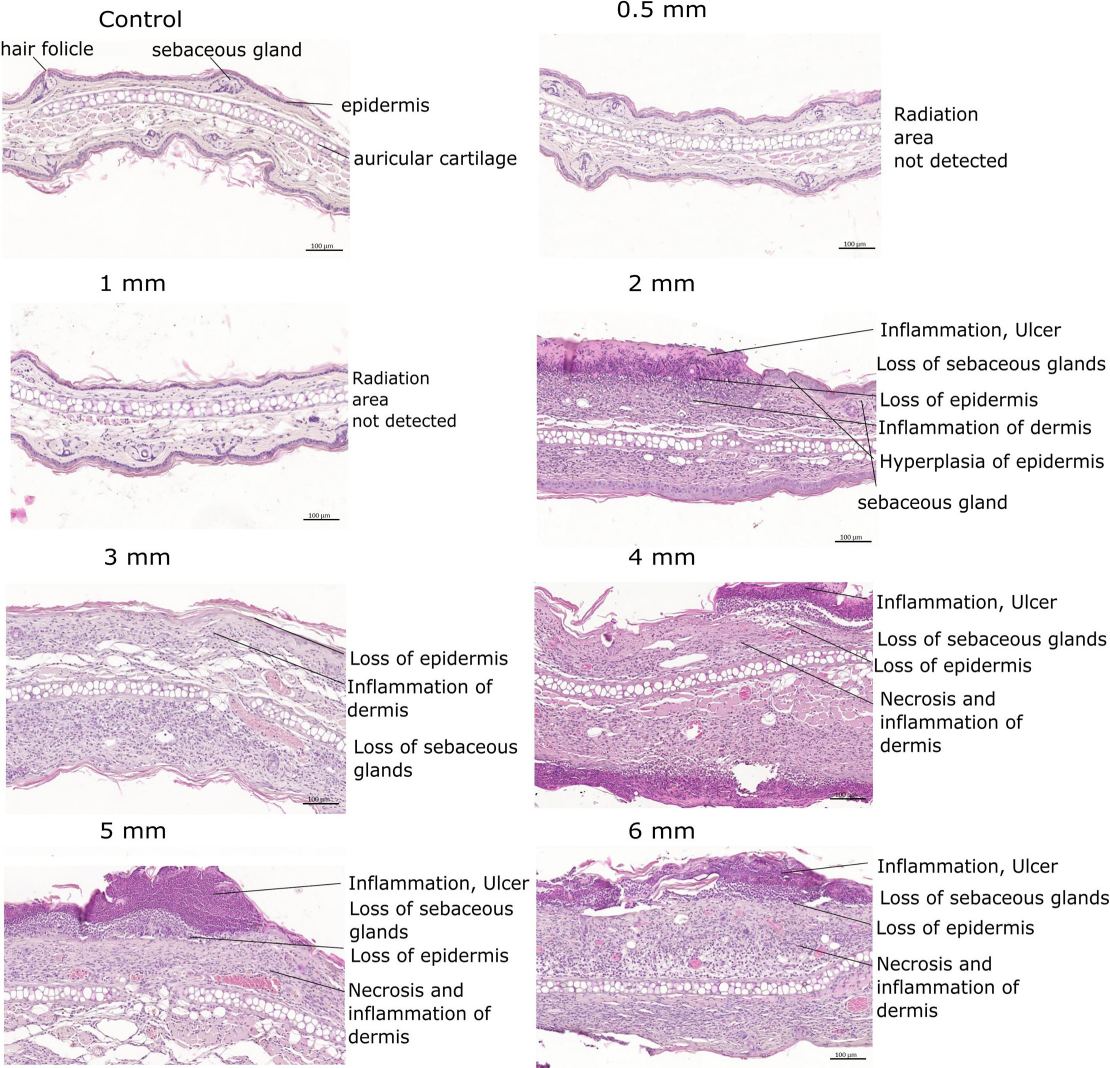
Representative H&E stained histological sections for each group at day 25.

To visualize the radiation effects on a cellular level, histological cross sections of 2 μm thickness were cut through the irradiated field of the ears dissected 25 days after irradiation, as the day of the expected maximum reaction according to the previous experiment of Girst et al. [8], and stained with hematoxylin and eosin. A representative section of every group including a control section of an untreated ear is shown in Fig 5.

The control ear shows a typical morphology with common skin elements such as hair follicles and sebaceous glands. While the two groups with the smallest irradiation fields (0.5 mm, 1 mm) could not be distinguished from the control ear, the larger fields yielded more definite reactions. To ensure that the sections were cut through the irradiation field, the Gafchromic films of the irradiation day were used as a first approximation. Ten sections were cut with a distance of 200 μm around the expected beam center. Strong signs of inflammation, ulceration and loss of sebaceous glands could be found in all groups with beam sizes larger than 2 mm. Hyperplasia of the epidermis as well as necrosis of the dermis were detected within the radiation fields as well. The histological findings are in agreement with the externally scored and measured endpoints. The measured ear swelling of the irradiated ears could be attributed to an accumulation of different cell types due to the induced immune response. The analysis of the Hematoxylin and Eosin (H&E) stained tissue samples did not yield any further information about specific cell types as well as healing processes involved.

## Discussion

Several techniques concerning spatially fractionated radiotherapy have recently been introduced and investigated for implementation into clinics. The higher dose tolerance of tissues irradiated with spatially fractionated beams could either reduce the side effects of radiotherapy or increase the implemented dose in the tumor, hence lead to a higher chance of cure. While several proof-of-principle experiments have already been carried out [4,5,8,7,14] showing the high potential of mini- or microbeam techniques, it is still crucial to find the optimum balance between healthy tissue sparing and (sufficient) tumor control.

The aim of the presented study was to compare different single pencil beam sizes applied in an *in-vivo* mouse ear model. Our study using clinically relevant beam diameters between 0.5 mm and 6 mm as well as a therapeutic dose of 60 Gy in all beams is able to deliver important information about the limits of spatially fractionated radiotherapy techniques. It is assumed that the outcomes are also valid for protons or heavier ions since a negligible amount of proliferating cells remain in a field irradiated with a 60 Gy photon dose and ions tend to have an even higher biological effectiveness. However, if the cell death pathways play an important role for the toxicity reaction, much higher doses, as they may occur in spatial fractionation techniques with tiny minibeams, or particles with different relative biological effectiveness and Gaussian beam shapes could influence the cell death and therefore the results.

A dose of 60 Gy applied in a field of 6 mm diameter leads to an almost 3-fold swelling of the ears and even to a 4.5-fold swelling with larger fields applied in a previous study [8]. The definite to severe skin reactions could be found in external scores (size measuring as well as visual scoring) down to a beam diameter of 3 mm with decreasing reactions for smaller beams. The 2 mm beam size showed no significantly observable scores, but had a slightly increased ear thickness. No ear swelling and barely skin score reactions were found for beams of 0.5 mm and 1 mm in diameter.

Even though a dose volume effect might be involved, the sharp limit of tissue sparing as suggested by Dilmanian et al. [7] could not be confirmed in this study. A general limit for minibeam sizes is hard to assess since the type of tissue being irradiated seems to play a crucial role. Curtis et al. found adverse effects in the irradiated brain of mice with deuteron beams of 25 μm size and doses of about 4000 Gy [17]. In comparison, the study of Girst et al. found no effects for mouse skin irradiated with proton minibeams of the size of 180 μm (sharp, squared) and doses as high as 6000 Gy [8], which is a factor of 66 larger irradiated field area as well as a factor of 1.5 higher dose. Neglecting the LET variation of 22 MeV deuterons (~ 4.32 keV/μm) to 20 MeV protons (~ 2.65 keV/μm) means that this huge difference is only attributable to the fact that different tissues were irradiated. Curtis also found a completely destructed cortex for 1 mm beams with an applied dose of 140 Gy [17]. However, no effects were visible in the histological sections in this study for 1 mm beam sizes. The study of Prezado et al. [14] supports our finding that a 1 mm (pencil) beam shows no radiation response, as they found a strong sparing effect in the irradiated brain for (planar) proton minibeam grids of 1.1 mm sized beams (3.2 mm beam distance) and a peak dose of 57 Gy. However, it needs to be added that both studies are under the dose limit of Curtis [17] and therefore still need clarification in further studies.

In the presented study, the mouse ear model seems to react in a smooth transition from no to severe radiation responses as observed by the quadratic increase of the ear swelling with beam diameter rather than a step function. In biological terms, the quadratic correlation with beam diameter implies that the radiation response is proportional to the amount of hit cells. The score of the radiation response remains below a significant reddening up to a beam diameter of 2 mm. This opens up the potential of spatial fractionation to at least such beam sizes. However, beam size limits may differ with tissue types since skin heals minibeam irradiated fields in a different way than, e.g. brain tissue due to the higher amount of proliferating cells, the migration of unaffected cells and maybe also a better immune or healing response in general.

While the skin reactions after high dose single beam irradiation were investigated in this study, a remaining question is the interaction of multiple minibeams in an irradiation field as well as the influence of ultra-high doses as they could also occur in minibeam therapy. One of the most crucial pieces of information will be the influence of the inter-beam distance in the used grid pattern on the healthy tissue sparing effects. Moreover, the sparing will likely be influenced by the sort of irradiated tissue. Thus, similar studies on other tissue types as well as varying doses are needed.

## Conclusion

This mouse ear study has demonstrated that a high dose (up to 60 Gy) irradiation with a single pencil beam in sizes of up to and including 1 mm is not causing swelling or any visible skin reactions in the mouse ear. Beam sizes of 2 mm and larger show a smooth transition to adverse side effects rather than a sharp response induction. As a consequence, these findings can be extrapolated towards the presence of some sparing effect of spatially fractionated beams larger than 1 mm, although sub-millimeter beams are preferable. If boundary conditions do not allow for sub-millimeter beams, spatial fractionation is still beneficial with reduced side effects in skin, muscle and cartilage tissue as they appear in mouse ears. The influence of the surviving cells surrounding the minibeams, thus the effect of a total minibeam pattern needs to be further investigated.

## Supporting information

S1 Table All measured and scored mouse data. Every measurement was repeated thrice and the scoring was performed under four eyes principle.
(XLSX)

## References

[1] Brauer-Krisch E, Bravin A, Lerch M, Rosenfeld A, Stepanek J et al (2003) MOSFET dosimetry for microbeam radiation therapy at the European Synchrotron Radiation Facility. Med Phys 30 (4): 583–589. 10.1118/1.1562169 12722810

[2] Prezado Y, Thengumpallil S, Renier M, Bravin A (2009) X‐ray energy optimization in minibeam radiation therapy. Medical physics 36 (11): 4897–4902. 10.1118/1.3232000 19994498

[3] Laissue JA, Blattmann H, Wagner HP, Grotzer MA, Slatkin DN (2007) Prospects for microbeam radiation therapy of brain tumours in children to reduce neurological sequelae. Dev Med Child Neurol 49 (8): 577–581. 10.1111/j.1469-8749.2007.00577.x 17635201

[4] Dilmanian FA, Rusek A, Fois GR, Olschowka J, Desnoyers NR et al (2012) Interleaved carbon minibeams: An experimental radiosurgery method with clinical potential. International Journal of Radiation Oncology* Biology* Physics 84 (2): 514–519. 10.1016/j.ijrobp.2011.12.025 22342299

[5] Zlobinskaya O, Girst S, Greubel C, Hable V, Siebenwirth C et al (2013) Reduced side effects by proton microchannel radiotherapy: study in a human skin model. Radiat Environ Biophys 52 (1): 123–133. 10.1007/s00411-012-0450-9 23271171

[6] Griffin RJ, Koonce NA, Dings, Ruud P M, Siegel E, Moros EG et al (2012) Microbeam radiation therapy alters vascular architecture and tumor oxygenation and is enhanced by a galectin-1 targeted anti-angiogenic peptide. Radiation research 177 (6): 804–812. 22607585 10.1667/rr2784.1 PMC3391740

[7] Dilmanian FA, Zhong Z, Bacarian T, Benveniste H, Romanelli P et al (2006) Interlaced x-ray microplanar beams: a radiosurgery approach with clinical potential. Proceedings of the National Academy of Sciences 103 (25): 9709–9714. 10.1073/pnas.0603567103 PMC1480471 16760251

[8] Girst S, Greubel C, Reindl J, Siebenwirth C, Zlobinskaya O et al (2016) Proton Minibeam Radiation Therapy Reduces Side Effects in an In Vivo Mouse Ear Model. International Journal of Radiation Oncology* Biology* Physics 95 (1): 234–241. 10.1016/j.ijrobp.2015.10.020 26692028

[9] Sammer M, Greubel C, Girst S, Dollinger G (2017) Optimization of beam arrangements in proton minibeam radiotherapy by cell survival simulations. Medical physics 44 (11): 6096–6104. 10.1002/mp.12566 28880369

[10] Withers HR, Taylor JMG, Maciejewski B (1988) Treatment volume and tissue tolerance. International Journal of Radiation Oncology* Biology* Physics 14 (4): 751–759. 10.1016/0360-3016(88)90098-3 3350731

[11] Källman P, Ågren A, Brahme A (1992) Tumour and normal tissue responses to fractionated non-uniform dose delivery. International journal of radiation biology 62 (2): 249–262. 1355519 10.1080/09553009214552071

[12] Dilmanian FA, Qu Y, Liu S, Cool CD, Gilbert J et al (2005) X-ray microbeams: Tumor therapy and central nervous system research. Nuclear Instruments and Methods in Physics Research Section A: Accelerators, Spectrometers, Detectors and Associated Equipment 548 (1): 30–37. 10.1016/j.nima.2005.03.062 PMC1828126 17369874

[13] Dilmanian FA, Qu Y, Feinendegen LE, Peña LA, Bacarian T et al (2007) Tissue-sparing effect of x-ray microplanar beams particularly in the CNS: is a bystander effect involved. Experimental hematology 35 (4): 69–77. 17379090 10.1016/j.exphem.2007.01.014

[14] Prezado Y, Jouvion G, Hardy D, Patriarca A, Nauraye C et al (2017) Proton minibeam radiation therapy spares normal rat brain: Long-Term Clinical, Radiological and Histopathological Analysis. Scientific reports 7 (1): 14403 10.1038/s41598-017-14786-y 29089533 PMC5663851

[15] Hubbel JH, Seltzer SM (1995) Tables of X-ray mass attenuation coefficients and mass energy-absorption coefficients from 1 keV to 20 MeV for elements: Z.

[16] Reinhardt S, Hillbrand M, Wilkens JJ, Assmann W (2012) Comparison of Gafchromic EBT2 and EBT3 films for clinical photon and proton beams. Medical physics 39 (8): 5257–5262. 10.1118/1.4737890 22894450

[17] Curtis HJ (1967) The use of a deuteron microbeam for simulating the biological effects of heavy cosmic-ray particles. Radiation Research Supplement 7: 250–257. 6058661

